# Tobacco Use and Its Association with Mental Morbidity and Health Compromising Behaviours in Adolescents in Indonesia

**DOI:** 10.31557/APJCP.2021.22.1.31

**Published:** 2021-01

**Authors:** Karl Peltzer, Supa Pengpid

**Affiliations:** 1 *Department of Research Administration and Development, University of Limpopo, Turfloop, South Africa. *; 2 *ASEAN Institute for Health Development, Mahidol University, Salaya, Phutthamonthon, Nakhonpathom, Thailand. *

**Keywords:** Tobacco use, mental health, health behaviour, adolescents, Indonesia

## Abstract

**Background:**

Limited evidence has been established on associations between tobacco use and mental morbidity and health compromising behaviours. The study aimed to investigate the associations between tobacco use, mental problems, and health risk behaviour among adolescents attending school in Indonesia. Methods: Nationally representative data were studied from 11,124 adolescents that took part in the cross-sectional “Indonesia Global School-Based Student Health Survey (GSHS) in 2015”.

**Results:**

The prevalence of current tobacco use was 12.8%. In adjusted logistic regression analysis, compared to non-current or never tobacco users, current tobacco use was associated with eight of eight mental problem indicators (lonely, anxiety, no close friend, suicidal ideation, suicide plan, suicide attempt and current alcohol use), two of four dietary risk behaviours (soft drink and fast food consumption) and seven of ten other health risk behaviours (in a physical fight, bullied, injury, ever sex, school truancy, and two sub-optimal hand hygiene behaviours).

**Conclusion:**

Compared to nontobacco users, current tobacco users had significantly higher mental problem indicators and health risk behaviours. Multiple comorbidity with tobacco use should be targeted in interventions.

## Introduction

Tobacco causes the death of 8 million persons per year (WHO, 2019). The majority of the world’s smokers (80%) reside in developing countries (WHO, 2019). Most users of tobacco initiate this habit when they are young during adolescence (Aldrich et al., 2014). More than one in ten (13.6%) adolescents in developing countries were current tobacco users (Xi et al., 2016). Smoking causes various diseases, including “cancer, heart disease, stroke, lung diseases, diabetes, and chronic obstructive pulmonary disease (COPD)” (CDC, 2018). Fewer studies have linked tobacco use with mental morbidity and health compromising behaviours. 

Some studies found a probable association between tobacco use and mental distress in young people (Chaiton et al., 2010; Lee et al., 2018; Peltzer and Pengpid, 2017; Saravanan and Heidhy, 2014), including suicidal ideation and suicide attempts (Han et al., 2009;

Järvelaid, 2004; Lee et al., 2018; Tomori et al., 2001). Several studies found a relationship between tobacco use and alcohol use (Fujita and Maki, 2018; Tomori et al., 2001; Wang et al. 2017), and illicit drug use (Zammit et al., 2018; Tomori et al., 2001). 

A number of investigations identified that compared to non-smokers, smokers engaged more likely in poor dietary behaviour, such as inadequate fruit and vegetable consumption (Wang et al., 2017; Lee and Yi, 2016), fast food consumption (Hrubá et al., 2010; Larson et al., 2007; Wang et al., 2017), higher frequency of eating out (Fujita and Maki, 2018), high sugar foods (Lee and Yi, 2016), soft drink intake (Larsen et al., 2007; Wang et al., 2017), high sodium or salty snacks consumption (Hrubá et al., 2010), were less likely to eat milk and dairy products (Lee and Yi, 2016; Wang et al., 2017), and skipped breakfast (Cohen et al., 2003; Wang et al., 2017). In addition, tobacco use increased the odds of exercise (Lee and Yi, 2016) and school truancy (Tomori et al., 2001). 

Studies are needed on the association between tobacco use, psychiatric morbidity, and health risk behaviour among adolescents in developing countries, such as in Indonesia, in which smoking is on the rise. The study aimed to assess the associations between tobacco use, mental problems, and health risk behaviour among adolescents in Indonesia.

## Materials and Methods

*Sample and procedures*

Cross-sectional nationally representative data from the 2015 GSHS in Indonesia were analysed (WHO, 2015a); sampling details were described previously (Pengpid and Peltzer, 2019). “*The Ethics Commission for Health Research and Development approved the study and informed consent was obtained from the participating schools, parents, and students.*”(WHO, 2015a).

*Measures*

*Outcome variables*

*Mental problem indicators*

Loneliness: “*mostly or always lonely during the past 12 months.” Anxiety: *”mostly or always been so worried about something that could not sleep at night in the past 12 months.”

No close friends: “*having no close friends.*” Suicidal behaviour: suicidal ideation, suicide plan, and suicide attempt in the past 12 months. Current alcohol use: “*≥1 days, at least one drink containing alcohol in the past 30 days.*” (WHO, 2015b).

*Dietary risk variables*

Soft drink consumption: drinking ≥1 time per day “*carbonated soft drinks, such as Coca-Cola, Sprite, Fanta, or Big Bola (excluding diet soft drinks).*” Inadequate fruit intake: “*<twice/day during the past 30 days.*” Inadequate vegetable intake: “*<3 times/day during the past 30 days*.” Fast food consumption: “*≥2 days/week eating food from a fast food restaurant.*” 

Inadequate physical activity: “*<7 days at least 60 minutes of moderate to vigorous-intensity physical activity*” (WHO, 2015b, 2017).

*Other health risk behaviours*

Sedentary behaviour: “*≥3 hours/day sitting when you are not in school or doing homework*” (Guthold et al., 2010). In a physical fight: any in the past 12 months. Being bullied: any day in the past 30 days. Serious injury: any during the past 12 months. School truancy: any day during the past 30 days. Ever sex: ever having had sexual intercourse. Inadequate tooth brushing: “*<2 times/day cleaning or brushing one’s teeth*.” Inadequate hand hygiene: “not always washing hands after toilet, before eating and with soap.” (WHO, 2015b).

Exposure variable

Tobacco use: “*During the past 30 days, on how many days did you smoke cigarettes/use any tobacco products other than cigarettes, such as sirih, piper betel, cerutu, or cigars?*” Responses ranged from “1=0 days to 7=All 30 days (coded 1=0 and 2–7=1)”

*Confounding variables*

Age, sex, and hunger (in the past month). Secondary smoke: any day in the past week.

Parental tobacco use: any parent or guardian. Peer support: “*exposure to kind and helpful students in school in the past month*” (never, rarely, or sometimes=low, and most of the time or always=high). Parental support: “*mostly or always parental supervision, parental connectedness, parental bonding, and parental respect for privacy in the past 30 days.*” (WHO, 2015b) (0=low, 2=moderate, and 3-4=high support).

*Data analysis*

Frequency, mean and standard deviations were calculated to describe the sample and its indicators. To test for differences in proportions, Pearson Chi-square tests were used. Logistic regression was used to determine the associations between tobacco use and mental problems and health compromising behaviours. Health outcomes found to have a significant association between tobacco use and health indicators were subsequently included in the multivariable logistic regression model, which was adjusted for relevant confounders (age group, sex, experience of hunger, secondary smoke, parental tobacco use, peer support, and parental support). Missing data were excluded from the calculations. P<0.5 was accepted as significant. “*STATA 15.00 (StatCorp LP, College Station, TX)*” was used for all statistical procedures, taking the complex study design into account.

## Results

*Descriptive Statistics*

The study sample consisted of 11,124 in-school adolescents (overall response rate 94%) (mean age 14.0 years, with a Standard Deviation of 1.6) in Indonesia, 51.1% were girls, 43.3% experienced sometimes or mostly or always hunger in the past month, 77.6% had exposure to secondary smoke in the past week, and 53.8% had parents who used tobacco. In addition, 39.1% of the students had mostly or always support by their peers, and 22.4% scored high on parental support. The prevalence of current tobacco use was 12.8%. Current tobacco use was significantly higher in males, older adolescents, those who experienced more frequent hunger, those that were exposed to secondary smoke, parental tobacco use, had low peer, and parental support (see Table 1).

*Associations between tobacco use and mental problems and health compromising behaviour*

In adjusted logistic regression analysis, compared to non-current or never tobacco users, current tobacco use was associated with eight of eight mental problem indicators [lonely: Adjusted Odds Ratio-AOR: 1.87, 95% Confidence Interval-CI: 1.37-2.54), anxiety (AOR: 1.95, 95% CI: 1.36-2.83), no close friend (AOR: 1.59, 95% CI: 1.08-2.36), suicidal ideation (AOR: 2.39, 95% CI: 1.58-3.62), suicide plan (AOR: 2.02, 95% CI: 1.53-2.68), suicide attempt (AOR: 6.96, 95% CI: 4.43-10.93) and current alcohol use (AOR: 14.97, 95% CI: 8.42-26.63)], two of four dietary risk behaviours [soft drink intake (AOR: 1.54, 95% CI: 1.31-1.83) and fast food consumption (AOR: 1.37, 95% CI: 1.14-1.65)] and seven of ten other health risk behaviours [in a physical fight (AOR: 2.85, 95% CI: 2.39-3.38), bullied (AOR: 1.87, 95% CI: 1.55-2.26), injury (AOR: 2.04, 95% CI: 1.68-2.47), school truancy (AOR: 2.45, 95% CI: 2.01-2.98), ever sex (AOR: 2.63, 95% CI: 2.05-3.38), not always washing hands after toilet (AOR: 1.29, 95% CI: 1.10-1.51) and not always washing hands before eating (AOR: 1.32, 95% CI: 1.09-1.60)] (see Table).

**Table 1:** Sample Characteristics of School-Going Adolescents in Indonesia, 2015 (N=11124)

Variables (#missing data)	Sample		Current tobacco use		p-value^1^
	N	%	N	%	
All	11,124		1,316	12.8	
Sex (#32)					
Female	6,020	51.1	148	2.4	<0.001
Male	5,090	48.9	1,156	23.6	
Age (#18)					
≤13	4,549	43.6	425	9.5	<0.001
14	2,565	24.1	300	12.5	
15	1,943	14.7	255	16.3	
≥16	2,067	17.6	329	18.1	
Experience hunger (#51)					
Never	4,944	44.7	503	11.2	0.002
Rarely	1,260	12	154	12.4	
Sometimes/mostly/always	4,887	43.3	649	14.5	
Secondary smoke: any day in the past week (#117)					
No	2,520	22.4	111	4.8	<0.001
Yes	8,505	77.6	1,191	15	
Parental tobacco use: any parent or guardian (#117)					
No	5,084	46.2	552	11.6	0.01
Yes	5,946	53.8	747	13.7	
Peer support (#165)					
Low	6,695	60.9	875	14.0	<0.001
High	4,282	39.1	412	10.6	
Parental support (#407)					
Low (0-1)	5,173	48	782	16.4	<0.001
Medium (2)	3,174	29.6	294	10.1	
High (3-4)	2,388	22.4	164	7.2	

^1^Based on Chi-square tests

**Table 2:** Associations between Tobacco Use and Health Outcomes

Health outcomes	UOR (95% CI)	AOR (95% CI)1
Mental problem indicators		
Lonely (past year) (6.2%)	1.79 (1.41, 2.28)***	1.87 (1.37, 2.54)***
Anxiety (past year) (4.6%)	2.24 (1.68, 2.98)***	1.95 (1.36, 2.83)***
Having no close friends (3.0%)	1.75 (1.27, 2.40)***	1.59 (1.08, 2.36)*
Suicidal ideation (past year) (5.2%)	2.28 (1.67, 3.12)***	2.39 (1.58, 3.62)***
Suicide plan (past year) (5.5%)	2.10 (1.58, 2.79)***	2.02 (1.53, 2.68)***
Suicide attempt (past year) (3.9%)	6.09 (4.30, 8.63)***	6.96 (4.43, 10.93)***
Current alcohol use (past month) (4.4%)	21.17 (13.14, 34.10)***	14.97 (8.42, 26.63)***
Dietary risk variables		
Soft drink (≥1 day) (27.9%)	1.59 (1.38, 1.83)***	1.54 (1.31, 1.83)***
Fruit consumption (<2 day) (64.3%)	1.04 (0.89, 1.22)	---
Vegetable intake (<3 day) (71.4%)	1.07 (0.93, 1.24)	---
Fast food (≥2 times/week) (25.5%)	1.41 (1.17, 1.73)***	1.37 (1.14, 1.65)***
Other health risk behaviours		
Physical inactivity (past week) (87.8%)	1.04 (0.81, 1.34)	---
Sedentary (≥3 hrs/day) (27.3%)	1.48 (1.22, 1.80)***	1.23 (1.00, 1.53)
In physical fight (past year) (23.4%)	4.10 (3.59, 4.69)***	2.85 (2.39, 3.38)***
Bullied (past month) (20.6%)	2.36 (1.95, 2.86)***	1.87 (1.55, 2.26)***
Serious injury (past year) (29.6%)	3.01 (2.57, 3.53)***	2.04 (1.68, 2.47)***
School truancy (past month) (20.1%)	3.10 (2.62, 3.66)***	2.45 (2.01, 2.98)***
Ever had sexual intercourse (5.3%)	3.26 (2.64, 4.02)***	2.63 (2.05, 3.38)***
Tooth brushing (<2 times/day) (10.8%)	1.84 (1.48, 2.28)***	1.10 (0.86, 1.42)
Wash hands after toilet (not always, past month) (38.3%)	1.55 (1.34, 1.80)***	1.29 (1.10, 1.51)***
Wash hands before eating (not always, past month) (47.8%)	1.52 (1.29, 1.80)**	1.32 (1.09, 1.60)**
Uses soap in washing hands (not always, past month) (60.7%)	1.44 (1.23, 1.69)***	1.12 (0.93, 1.35)

UOR, Unadjusted Odds Ratio; AOR, Adjusted Odds Ratio; ^1^Adjusted for age, sex, experience of hunger, secondary smoke, parental tobacco use, peer support, and parental support

## Discussion

In this large nationally representative study of school-going adolescents in Indonesia, compared to non-current or never tobacco users, current tobacco users had significantly poorer mental health (lonely, anxiety, no close friends, suicidal ideation, suicide plan, suicide attempts and current alcohol use) and increased odds for several health compromising behaviours (soft drink intake, fast food consumption, in a physical fight, bullied, injury, school truancy, ever sex, and sub-optimal hand hygiene behaviours) than non-current or never tobacco users in adjusted analysis. These results are generally in line with previous findings (Chaiton et al., 2009; Fujita and Maki, 2018; Halperin et al., 2010; Han et al., 2009; Hrubá et al., 2010; Larson et al., 2007; Lee et al., 2018; Peltzer and Pengpid, 2017; Saravanan and Heidhy, 2014; Tomori et al., 2001; Wang et al., 2017). Alcohol use is known to be a comorbidity of tobacco use (Konkolÿ et al., 2016; Peltzer and Pengpid, 2018), and this study showed a very high association between tobacco and alcohol use. It is possible that tobacco users that are likely to deny the harms of tobacco use are also likely to deny the risks of other health risk behaviours (Zammit et al., 2018). Tobacco use may be utilized by students to cope with psychosocial problems, as a form of “self-medication” (Mathew et al., 2017; Tomori et al., 2001). The finding may be supported by the study results of very high comorbidity of tobacco use with alcohol use.

Some studies (Wang et al., 2017; Lee and Yi, 2016) found an association between tobacco use and physical activity and inadequate fruit and vegetable intake, but we identified no significant associations in this study. Study findings support the importance of understanding the various health risk behaviours tobacco users are more likely to engage in for the development of school mental and physical health promotion (Pengpid and Peltzer, 2020). This study appears to confirm that tobacco use among adolescents is linked to the development of mental and physical health risk factors (Tomori et al., 2001). Programmes should not only target tobacco use cessation but also promote a variety of healthy behaviours, in an integrated school health promotion programme.

*Study limitation*

Due to the cross-sectional design of the study, we are unable to determine the direction of the relationship between tobacco use and health indicators. We can also not generalize the findings to adolescent in Indonesia, since only school-going participants were included in the study. Out-off school adolescents may have a different pattern of tobacco use associations. Due to the self-report of the data, it is possible that some of the health and mental health indicators were underreported.

In conclusion, in this large cross-sectional national study in Indonesia, compared to non-current or never tobacco users, current tobacco users had significantly poorer mental health (lonely, anxiety, no close friends, suicidal ideation, suicide plan, suicide attempts and current alcohol use) and increased odds for several health compromising behaviours (soft drink intake, fast food consumption, in a physical fight, bullied, injury, ever sex, school truancy, and sub-optimal hand hygiene behaviours) than non-current or never tobacco users in adjusted analysis. Multiple comorbidities with tobacco use should be targeted in interventions.
